# Hearing health literacy among professional and amateur musicians

**DOI:** 10.1038/s41598-024-79875-1

**Published:** 2024-11-18

**Authors:** Eva Schurig, Robin Hake, Michael Birke, Deborah Derks, Kai Siedenburg, Gunter Kreutz

**Affiliations:** 1 Department of Music, Carl von Ossietzky Universität Oldenburg, Oldenburg, Germany; 2 Department of Medical Physics and Acoustics, Carl von Ossietzky Universität Oldenburg, Oldenburg, Germany; 3 Signal Processing and Speech Communication Laboratory, Graz University of Technology, Graz, Austria

**Keywords:** Hearing health, Musicians, Professional, Amateur, Health literacy, Occupational health, Disease prevention, Risk factors, Human behaviour

## Abstract

Musicians create sound that is enjoyable to themselves and their audience, but this same sound also threatens their hearing health due to high sound pressure levels generated by their own and their fellow musicians’ instruments. Here we seek to identify musicians’ hearing health awareness in relation to their coping strategies. 370 professional and 401 amateur musicians in total responded to a questionnaire on hearing health, which included quantitative elements and open-ended questions. Findings reveal that musicians are generally aware of hearing health risks but also indicate a lack of discussion on that topic. However, inasmuch as respondents consider hearing protection as necessary, they articulate justified concerns about its potential impact on quality of performance and musical communication. In sum, musicians show literacy regarding their hearing health, but more guidance and training is needed to ensure effective protective measures.

## Introduction

Previous work suggests that professional musicians are exposed to excessive sound pressure levels during rehearsals and concerts^1–4^ as well as during their solitary practice^5^. A similar problem arises in amateur orchestras, albeit with lower exposure dosages compared to professionals^6^. Despite calls for measures to protect musicians’ hearing health, it seems that high sound pressure levels are still a discomforting part (in terms of hearing health) of the reality of playing music in orchestras for many musicians. Moreover, the problem could in part be masked by research demonstrating enhanced auditory processing skills of musicians, which points to the potential benefits of playing music for hearing health^7^.

Both professional and amateur musicians are at risk of suffering hearing damage^6^. This phenomenon has been studied with some emphasis on the former group, showing, for example, that there is a relationship between number of years in the profession and the accumulation of hearing health issues, such as hearing loss, tinnitus, or hyperacusis^1^. A historical cohort study discovered that musicians are almost four times as likely than the general population to have noise-induced hearing loss, and suffer from tinnitus at a higher rate as well^8^. Even among healthy early-career musicians, whose hearing does not differ from non-musicians, complaints of tinnitus, hyperacusis and hearing-in-noise difficulties are prevalent^9^. Studies with musicians based at distinct geographic locations have investigated and confirmed an elevated prevalence of hearing damage^10–15^. A systematic review^16^ revealed that 38.6% of professional musicians suffer from hearing loss to some degree while 26.3% have tinnitus. This is valid for jazz, rock and pop musicians as well as classical musicians^17^. The numbers concerning individual hearing protection uptake, however, reveal that even though many musicians are affected by hearing damage, only a small number of them wear hearing protection regularly^10,13,15,18,19^. Other studies point towards higher numbers of musicians that use hearing protection, for instance, 56% (of 44 participants)^17^ or 77.5% of early-career musicians^20^. Numbers in Australia appear to be higher as well (64%^5^), which the authors ascribe to a country-wide hearing conservation strategy^21^.

Research has also addressed preventative measures to protect musicians’ hearing health in response to the high prevalence of hearing issues in that group^5^. One group of such measures builds on controlling the sound sources, for example, by screens, mutes, or sitting further apart, whereas an alternative strategy favors individual hearing protection. Wenmaekers and colleagues^22^ discovered that the physical measures were less practical to effectively reduce sound pressure levels. Therefore, individual hearing protection is recommended.

Nevertheless, despite the positive effects of individual hearing protection, many musicians struggle with it even after long years of use^21^. The main reasons for not using individual hearing protection is the impact on performance, social pressure, and a lack of concern^20^. Couth and colleagues^20^ acknowledge that there are fundamental flaws with this kind of hearing protection that need be addressed before musicians are comfortable with using it regularly. An interview study with musicians^23^ showed that the use of ear plugs is perceived ambivalently: whereas limitations include reduced ability for pitch intonation and sound balance, ear plug use is also perceived to yield benefits, such as reduced susceptibility to pain and auditory fatigue effects. Particularly younger musicians claim that earplugs even improve the sound clarity in ensembles^23^. A recent study with US musicians^24^ discovered several factors that might facilitate the current use of hearing protection among professional musicians, namely previous use of hearing protection, owning fitted hearing protection and having hearing issues. The main barrier hampering the use of hearing protection was its inconvenience particularly during rehearsals and concerts. Interestingly, string players reported wearing hearing protection after a diagnosed hearing damage^18^, whereas percussionists use it as a precaution^18,24^.

### Hearing health literacy

Health literacy can be defined as “the knowledge, motivation and competences to access, understand, appraise and apply health information in order to make judgments and take decisions in everyday life concerning health care, disease prevention and health promotion to maintain or improve quality of life throughout the course of life”^25^, but it has also been described as a social practice that depends on the situation, the available resources and the person’s own experiences and knowledge^26^. With regards to hearing in particular, Piao and colleagues^27^ found that individuals with hearing impairments generally tend to show lower health literacy, possibly due to reduced communication with health providers and less understanding of medical services. Similarly, Wells and colleagues^28^ note that hearing aid use is linked to higher health literacy due to an increased ability to communicate with health providers and thus the availability of health information.

### Health literacy among musicians

Guptill and colleagues^29^ developed the Musicians’ Health Literacy Questionnaire and found an overlap between general health literacy and amateur musicians’ health literacy. Overall, there seems to be a low health literacy among musicians, which might also be the case because health programs for musicians are still rare among music institutions and conservatories^30^. The lack of health education in tertiary education is also reflected in music students’ low awareness of health and fitness with regard to playing their instrument^31^. Music students tend to accept pain while making music as inevitable and are less knowledgeable about injury prevention than professional musicians^31^. An earlier study also revealed that orchestra musicians believe that being injured reflects on their talent and impacts their employability^32^. Finally, they report high pressure to perform and, particularly in orchestral cultures where there is a negative attitude towards injury, tend to ignore physical pain and hide injuries from their peers and teachers^32^.

To counteract this lack of knowledge and following recommendations of prior research^30^, Matei and colleagues^33–35^ carried out training sessions with musicians on the topic of health education and discovered that participants’ knowledge on health issues was increased after the sessions^33,34^.

### Aims and research question

Hearing health is a critical issue among musicians around the world. In this paper, we therefore aimed to identify both professional and amateur musicians’ awareness of the potential hearing health issues related to their activities, what they do to counteract the risks associated with high sound pressure levels, and where musicians might know about risks but decide not to act on this knowledge.

## Results

### Quantitative

**Fig. 1:**
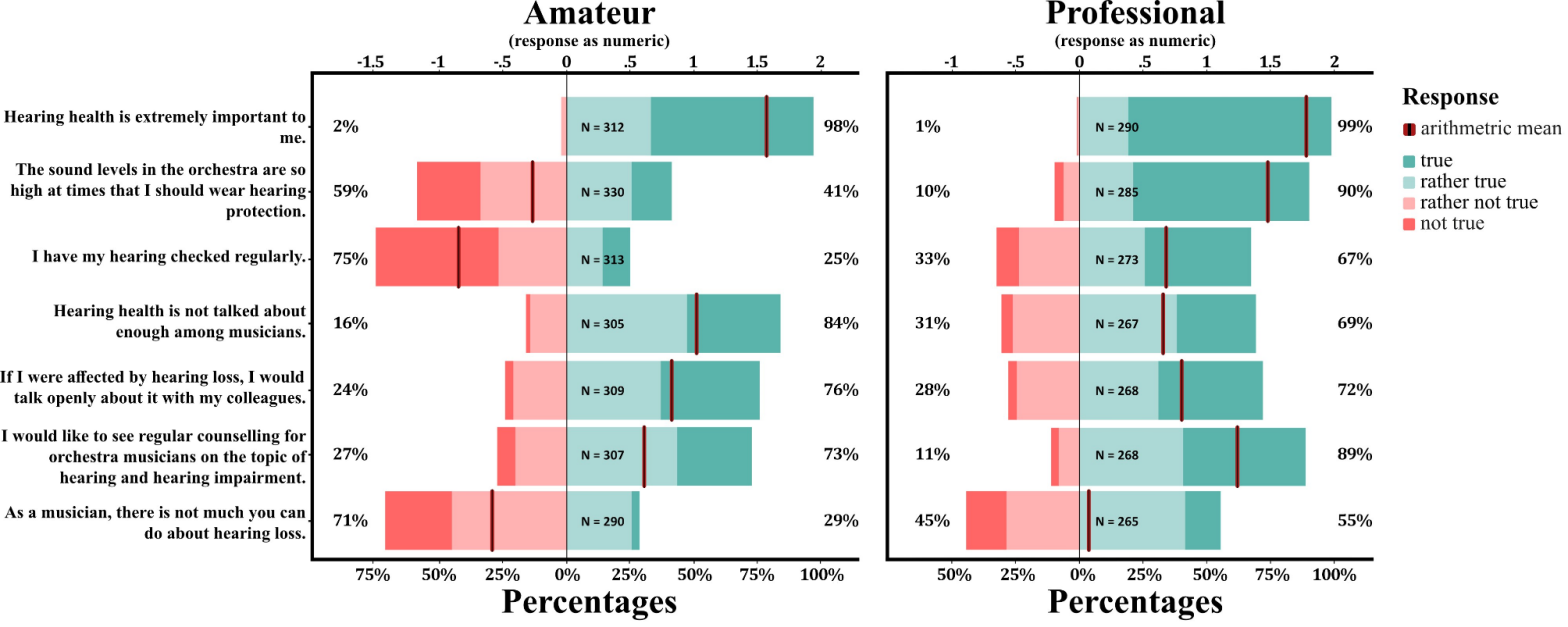
Perceptions of Hearing Health Among Musicians. This figure presents Likert scale responses from amateur (left panel) and professional (right panel) musicians regarding various statements about hearing health. Responses range from negative (“not true” and “rather not true”, in red) to positive (“rather true” and “true”, in green ). Note: ‘NA’ values are omitted.

Amateur and professional musicians both reported that hearing health was extremely important to them (98% of amateurs and 99% of professionals indicated at least a ‘rather true’ sentiment, as depicted in Fig. 1). Yet, professionals stated getting their hearing checked more regularly than amateurs (25% amateurs and 67% professionals, *M*_*diff*_ = − .88, *W* = 19358, *p* < .001, with a moderate effect size (*r*_*rb*_ = − .425)). Moreover, a significantly larger proportion of professionals acknowledge the necessity of wearing hearing protection as a safeguard against the loud sound levels prevalent in orchestras (90.2% compared to 41.2% amateurs replied with either ‘rather true’ or ‘true’, *M*_*diff*_ = -1.73, *W* = 17108, *p* < .001, *r*_*rb*_ = − 0.515). However, the adoption of hearing protection among musicians, particularly amateurs, remains low. Only 9.7% (33 out of 306) of amateurs and 58% (170 out of 292) of professionals reported utilizing hearing protection at all. Furthermore, among those who reported using hearing protection, the majority (76% of amateurs, 75% of professionals) admitted to not using it regularly during individual practice sessions. Hearing protection sees wider adoption during collaborative practices or concert performances, with 62% of professionals and 58% of amateurs acknowledging to utilize hearing protection ‘at least sometimes’.

Despite the widespread recognition of the importance of hearing health among musicians, a substantial majority of respondents, both among amateurs (84%) and professionals (69%), acknowledge that the topic is infrequently discussed within their community. Notably, while slightly more amateurs than professionals endorse this belief (*M*_*diff*_ = 0.38, *W* = 46506, *p* < .01, with a small effect size *r*_*rb*_ = 0.11), significantly more professionals express a desire for regular counseling on the subject of hearing health and hearing impairment (*M*_*diff*_ = − 0.56, *W* = 30820, *p* < .001, with a small effect size *r*_*rb*_ = − 0.2). This desire for guidance is prevalent in both groups, though, with 73% of amateurs and 89% of professionals indicating a preference for such support.

Professionals concede more frequently than amateurs that as musicians, there is not much one can do for mitigating hearing loss (29% of amateurs compared to 55% of professionals affirming a ‘rather true’ sentiment; *M*_*diff*_ = 0.57, *W* = 27096, *p* < .001, *r*_*rb*_ = − 0.23). Consequently, in total, 42% of respondents expressed some degree of resignation regarding their ability to influence their hearing health positively. Additionally, roughly a quarter of musicians exhibit reluctance to openly discuss any experienced hearing impairment among their colleagues (24% amateurs, 28% professional; *M*_*diff*_ = 0.05, *W* = 41616, *p* = .91, *r*_*rb*_ = 0).

### General hearing status

Questions regarding their general hearing status were answered by 319 amateur and 282 professional musicians. A substantial portion of these respondents reported having had a formal hearing assessments: 96% of professionals (N_total_ = 282) and 69% of amateurs (N_total_ = 319) stated having had their hearing checked before and reported the results of their hearing assessment (see Table 1). On average, the last hearing assessment for amateurs was 5 years and 3 months ago (SD = 6.5 years; N_total_ = 204) whereas, for professional musicians, it was 3 years (SD = 4 years; N_total_ = 255). Most of the respondents reported normal hearing, while some report mild and moderate hearing impairments (see Table 1 for more details). The remaining musicians, i.e., those without prior hearing assessment, estimated their hearing abilities by rating them according to descriptions of typical daily situations involving their hearing, for instance, “Yes, I have the feeling of being severely hearing impaired (hearing loss of 61–80 dB): I can barely understand speech without hearing aids and use lip-reading/sign language to assist communication.” Whereas one professional musician reported a severe hearing impairment, most amateur and professional musicians reported normal hearing or mild hearing impairment. There was no significant difference between amateur and professionals regarding hearing loss (*p* = .41). Furthermore, 69 amateurs (N_total_ = 318) and 72 professionals (N_total_ = 276) reported problems with tinnitus.

**Table 1:** Table 1.

	amateurs (319)	professionals (282)
audiometric test overall	220 (69%)	270 (96%)
normal hearing	*141 (64%)*	*172 (64%)*
mild HI	*40 (18%)*	*53 (20%)*
moderate HI	*15 (7%)*	*13 (5%)*
severe	*2 (1%)*	*0*
profound	*0*	*2 (1%)*
I don’t know	*17 (8%)*	*26 (9%)*
not specified	*5 (2%)*	*4 (1%)*
**self-reported hearing abilities overall**	**99 (31%)**	**12 (4%)**
normal	*60 (61%)*	*4 (33%)*
mild	*33 (33%)*	*4 (33%)*
moderate	*0*	*1 (8%)*
severe	*0*	*1 (8%)*
I don’t know	*3 (3%)*	*1 (8%)*
not specified	*3 (3%)*	*1 (8%)*

Note: Self-reported results of the audiometric tests as well as self-reported hearing abilities of professional and amateur musicians. “Not specified” indicates that respondents skipped this question. Respondents who did not remember the results of their audiometric tests were not asked to subsequently self-report their hearing abilities.

### Music-related hearing problems

**Fig. 2:**
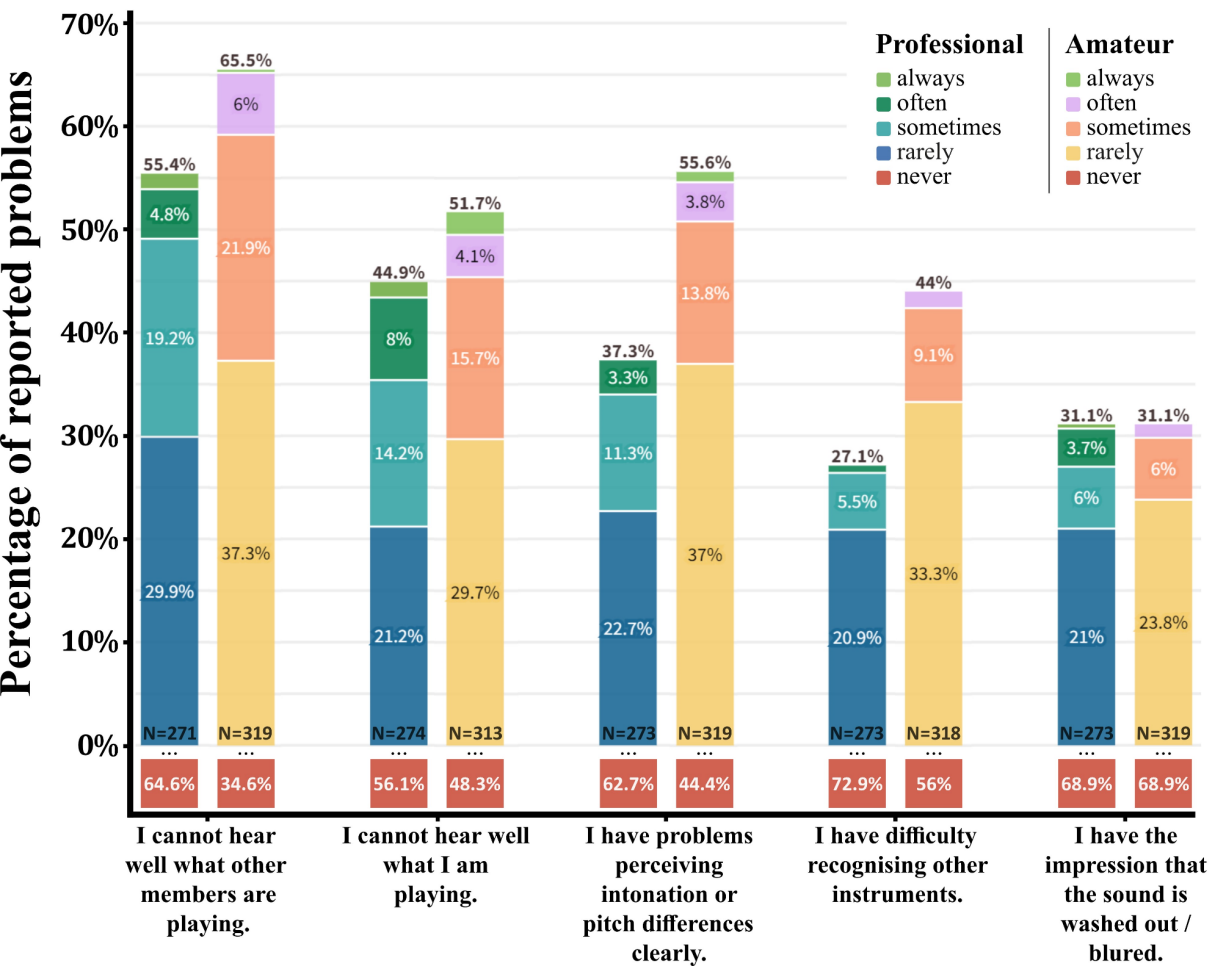
Reported Prevalence of Music-Related Hearing Problems. Data from professionals (left bars) and amateurs (right bars). See the legend for response categories. Note: ‘NA’ values were omitted, and responses below 3% are not explicitly displayed.

In addition to general hearing issues, musicians frequently encountered various music-related hearing difficulties. A notable proportion experienced challenges in hearing other orchestra members (55.4% of professionals versus 65.5% of amateurs reported experiencing this ‘rarely’ or more often) or themselves (ranging from 44.9 to 51.7%) while performing (refer to Fig. 2). Moreover, perceiving differences in intonation or pitch presented issues for a significant portion of respondents (37.3% of professionals versus 55.6% of amateurs). Some participants also struggled with recognizing different instruments (27.1% of professionals versus 44% of amateurs). Interestingly, the least frequently reported problem was a perception of washed-out or blurred sound (31.1% for both professionals and amateurs). Notably, amateurs consistently reported a higher prevalence of music-related hearing issues compared to professionals. However, statistical significance was observed only for difficulties in perceiving intonation or pitch differences (*M*_*diff*_ = 0.25, *W* = 50952, *p* < .001, with a small effect size *r*_*rb*_ = 0.14), challenges in recognizing other instruments (*M*_*diff*_ = 0.22, *W* = 50828, *p* < .001, with a small effect size *r*_*rb*_ = 0.15), and hearing what other members are playing (*M*_*diff*_ = 0.11, *W* = 47052, *p* < .05, with a very small effect size *r*_*rb*_ = 0.07).

**Fig. 3:**
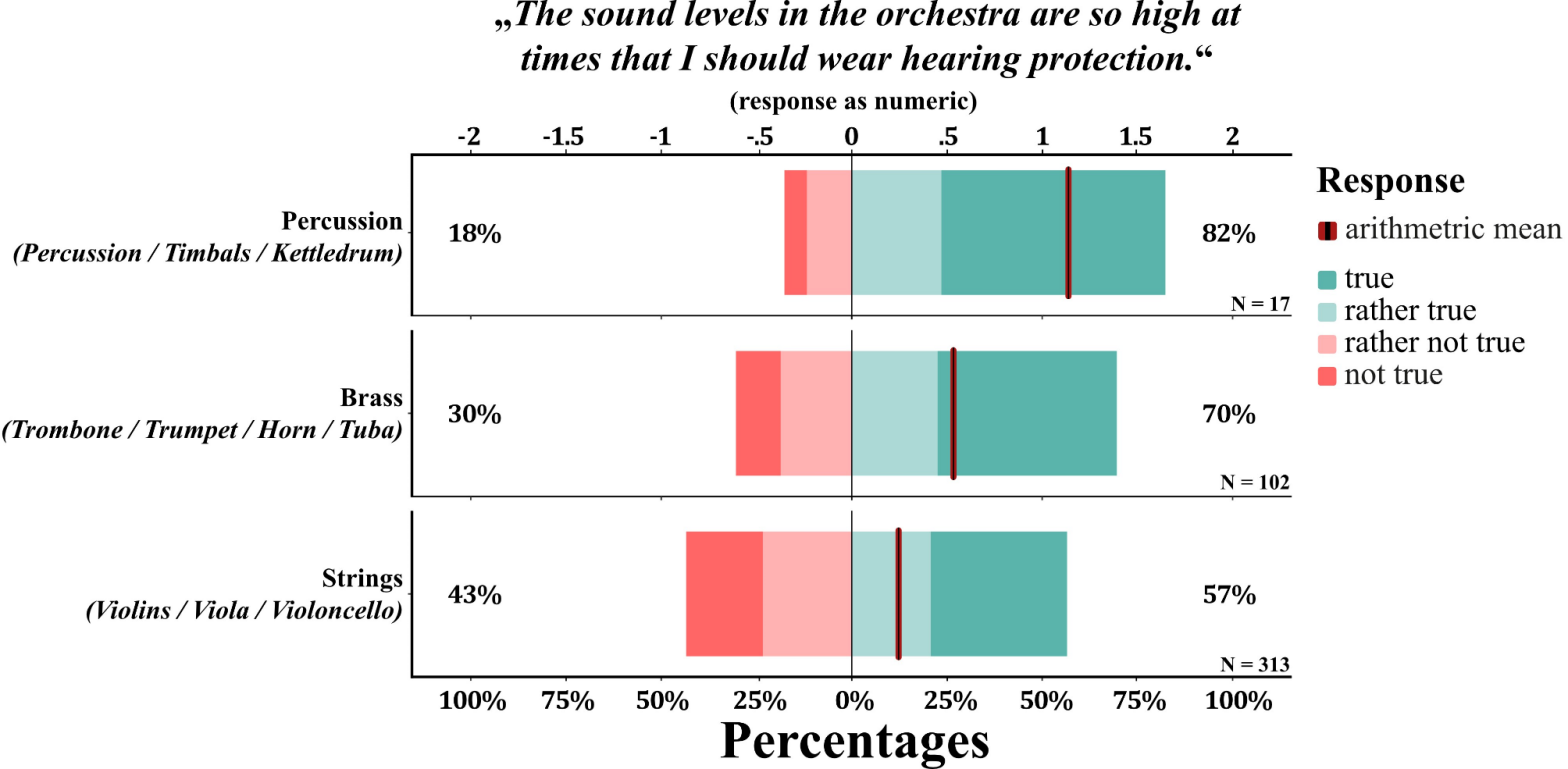
This figure illustrates Likert scale responses to the statement ‘The sound levels in the orchestra are so high at times, that I should wear hearing protection,’ categorized by the primary instrument of musicians. Responses were grouped by percussionists, brass players, and string players, with data from both professional and amateur musicians combined. The responses range from negative (‘not true’ and ‘rather not true’, depicted in red colors) to positive (‘rather true’ and ‘true’, green colors). Note: ‘NA’ values are omitted.

When examining the musician cohort categorized by instrument groups (considered irrespective of professional or amateur status), distinct variations emerged in the perceived importance of wearing hearing protection. Notably, when respondents were asked about the potential risk posed by orchestra sound levels to their hearing health, the majority of percussionists (72%), brass players (65%), but only half of the string players (50.5%) affirmed this concern. This pattern persisted when musicians were asked to evaluate the statement “*The sound levels in the orchestra are so high at times*,* that I should wear hearing protection*”. While only 57% of string players concurred with this statement (i.e., respond with “rather true” or higher), a higher proportion of brass players (70%) and percussionists (82%) acknowledged the necessity of protective measures (see Fig. 3). Upon applying Bonferroni correction for multiple comparisons, a significant difference was observed solely between string players and brass players (*M*_*diff*_ = 0.46, *W* = 18480, *p* = .04, *r*_*rb*_ = 0.11), while the difference between string players and percussionists was not statistically significant (*M*_*diff*_ = 0.89, *W* = 3474, *p* = .08, *r*_*rb*_ = 0.11). This outcome may be attributed to the conservative nature of the statistical test, which accounts for the relatively small sample size and the inherent variability among percussionists. Regarding actual hearing protection usage, string players (26.3%, 85 out of 323) and brass players (29.8%, 31 out of 104) reported rather similar rates of occasional use. Percussionists demonstrated the highest usage at 64.7% (11 out of 17). Interestingly, among those employing hearing protection, 57% of brass players and 62.4% of string players never used them during solo practice, in contrast to consistent use reported by all percussionists.

### Qualitative

In the qualitative analysis, all responses to open questions were considered no matter whether the participant was included in the quantitative analyses or not. The open responses of professional as well as amateur musicians showed that positive experiences with hearing protection were relatively rare (44 of 621 participants; 7%). Specifically, respondents stated that adjustments to the hearing protection could take some time, but once they were used to it, they embrace it. Avoidance of hearing issues and experiencing greater relaxation were mentioned as important reasons. Some of them also reported wearing the protection in certain situations only.

“Works well. Put it on for loud passages and take it off again for quiet ones.” (professional, male, 50–59 years old, trumpet, no. 1174).

“Whenever I wear hearing protection, I am much more relaxed and less stressed after rehearsals compared to no hearing protection.” (professional, female, 30–39 years old, 2nd violin, no. 1229).

However, the majority of respondents commented on hearing protection more negatively (692 of 2454 responses, 28%). Respondents claimed that wearing hearing protection could impair their performance, that they hear themselves differently, and cannot adjust their volume, intonation and sound to others.

“as if you were driving at a high speed through thick fog“ (professional, male, 50–59 years old, viola, no. 1513).

An additional issue concerns the intelligibility of speech either among musicians or the conductor.

“Additionally, I’m afraid that I won’t be able to hear the verbal instructions of the conductor and the conversations with colleagues.” (amateur, female, < 30 years old, 1st violin, no. 4382).

Furthermore, some of the respondents report that they use hearing protection even though it appears to be uncomfortable, impractical, and does not adapt to loud and quiet passages. Wind players can feel a heightened perceived inner pressure in the head in addition to sounds that originate from their playing (lips, tongue) which, so they claim, prevents them from using hearing protection.

“The inner pressure when blowing into the oboe is very intense, so that hearing protection is impedimental.” (professional, male, 60–69 years old, oboe, no. 1467).

“It’s uncomfortable to wear for long periods of time.” (professional, female, 30–39 years old, horn, no. 1500, original in English)

“The vibrations of the lips when producing each tone on the trombone cause strong additional sounds when using hearing protection.” (professional, male, 50–59 years old, trombone, no. 1090).

As opposed to many of the professional musicians, who feel that hearing protection is necessary but it impairs their performance or is uncomfortable, a number of amateur musicians stated that hearing protection is not necessary in an amateur orchestra, because high sound pressure levels are seldomly reached in their ensemble. This is also reflected in the category “no knowledge/experiences with hearing protection” as one of the reasons why participants did not use hearing protection. This category contained answers from 57 respondents, most of which (56) were amateur orchestra musicians. They claim that they had not thought about the topic of hearing protection before and/or were not aware that it might be important to use hearing protection in the orchestra. Interestingly, some of them write that nobody else has provided any information on this topic which is why they were unaware of it.

“Never thought about it.” (amateur, female, 30–39 years old, 2nd violin, no. 4788).

“Was never offered hearing protection; professional hearing protection if often very expensive.” (amateur, male, < 30 years old, bassoon, no. 5183).

Thus, these observations suggest that some amateur musicians tend to hold other people responsible for their hearing health, blaming them for not receiving enough information about the issue. In some cases, professional musicians behave similarly in shifting the responsibility towards medical professionals, the orchestra management or acousticians, all of whom do not seem to be able to help or understand the difficulties or specific requirements musicians face or need. They claim that their employer either does not provide any appropriate hearing protection or does not listen to complaints regarding that topic. Some of the professional musicians would have wanted to be informed about this topic, possibly already during their studies, and do not feel supported and heard which leads to frustration especially in combination with already existing hearing difficulties.

“Unfortunately, I was not informed about hearing protection at work on time. I had 3 instances of acute hearing loss and now my right ear is deaf …” (professional, female, 60–69 years old, 2nd violin, no. 4558).

“I feel left alone and misunderstood regarding this issue.” (professional, female, 50–59 years old, bassoon, no. 1460).

While many of the professional musicians struggle to get their employers to acknowledge hearing difficulties as more than an individual issue, the topic is also controversially discussed among colleagues. One musician states that there is “slow progress, but even young trumpet players seldomly understand the exposure [to high sound pressure] of the colleagues in front of them” (professional, male, 50–59 years old, viola, no. 1513). Several of the musicians (34) even explain that the topic of hearing health is still stigmatized in orchestras: people wearing hearing aids are seen as “inferior” (professional, male, 40–49 years old, bass, no. 1866), “weak, should not play in an orchestra, should resign” (professional, female, 50–59 years old, bassoon, no. 2139), they are less respected (professional, female, 40–49 years old, horn, no. 2255) and wearing hearing aids can be understood as “disregard of the musical achievement of colleagues” (professional, male, 30–39 years old, horn, no. 1120). One example of this stigmatization can be found in the following words of a professional musician: “Orchestral music is played and felt. A loud passage is different in character from a quiet one. This is intended by good composers. If someone has an issue with this, they should, during their training, have thought about getting a desk job instead.” (male, 60–69 years old, horn, no. 2331). However, it should be noted that this statement demonstrates one of the most extreme attitudes captured in the present dataset. Other responses reflect the perspective of someone who either wears hearing protection and feels judged by their colleagues or would like to have an open discussion on this topic among colleagues and feel that this is not possible. Only 5 respondents state that stigmatization is not an issue in their ensemble, and that hearing health is important for their peer group of colleagues.

## Discussion

The main aims of this study were to characterize hearing health awareness of professional and amateur musicians, their actions to counteract potential health issues related to their musical activities, and instances in which they might decide not to take preventive actions despite knowing about risks. We analysed quantitative and qualitative accounts of musicians and both sources of information turned out to yield novel insights into the perceived benefits and risks of wearing hearing protection in the orchestra, and thus also inform about the musicians’ hearing health awareness and literacy.

Most musicians in our study feel that hearing health is important and particularly professional musicians are aware of the potential health issues related to playing in an orchestra. This corroborates previous findings that professional musicians are more often exposed to high sound pressure levels than amateurs and therefore feel that they need to care for their hearing health^6^. Despite their positive attitude towards hearing protection, many of the participants are already affected by issues such as general hearing impairments, tinnitus or difficulties hearing other orchestra members. In fact, similar rates of hearing loss and tinnitus were discovered among the musicians in our study as compared to previous studies^16^. While several of the respondents aim to prevent these issues, professionals get their hearing checked more regularly than amateurs and wear hearing protection more often as well, although hearing protection is generally not used frequently. Similarly, other studies also point towards a low number of musicians wearing hearing protection regularly^10,18^. Amateurs might be less exposed because they do not rehearse as often compared to professionals, however, this does not mean that their hearing is not affected by their musical practice^4^. Further research on hearing health of amateur musicians is necessary to obtain a clearer picture of their music-related hearing problems.

Interestingly, the quantitative analysis discovered that brass players feel they should protect their hearing but rarely do while percussionists and string players wear hearing protection according to their sense of risk (high for percussionists, lower for strings). This finding agrees with previous research pointing out that percussionists wear hearing protection as a precaution^18,24^. There remains the question as to why there is this difference in sense of risk and actual protective behaviour between different groups of instruments in the orchestra. This should be explored in further studies.

As opposed to results by Couth and colleagues^20^, respondents of our study did not fail to use hearing protection due to a lack of concern but rather more differentiated underlying issues such as technical difficulties, lack of guidance and social constraints. Despite their knowledge on the possible health issues they face, particularly professional musicians feel limited in what they can do about it, which leads to frustration and resignation with regard to hearing protection. Many of the musicians feel they have to live with the situation as it is, even though their hearing health already is affected. That is, the professional musicians in this study are mostly literate regarding their hearing health, but this literacy also helps them to see the limitations of prevention strategies (technical issues, stigmatization, little support from the orchestra management, inability of acousticians to guide musicians).

Based on these results, we recommend the following possible actions:

- Despite the described practice of some of the respondents who use hearing protection only for loud passages, based on the literature^36^, hearing protection should be worn all the time to allow for an acclimatization effect. This also corresponds with descriptions of other participants who mentioned that it takes time to get used to hearing protection.
- Because many respondents reported being dissatisfied with either their hearing aids or their hearing protection in the context of music making, we recommend collecting and communicating the experience reports of those musicians who successfully use hearing aids or hearing protection.
- Not only should musicians be informed about possible implications of their musical practices on their hearing, the orchestra management needs to be made aware of the issues, and solutions should be offered to them based on current research. With this information, the management would be then able to fully support the musicians.
- Wind players and other musicians being affected by the occlusion effect when wearing hearing protection should be made aware of possible alternative options to hearing protection that prevent occlusion (e.g., earplugs with deep seals^37^).

One of the limitations of the present study is the possibility of a self-selection bias in that participants might have taken part who were more interested in this topic. Therefore, answers to questions regarding the importance of being aware of one`s hearing health would be answered affirmatively more often. Respondents with hearing issues will necessarily have dealt with this topic before and thus are likely to be more interested in the survey as well. This bias might be reduced by using a random-sampling strategy in future studies. Additionally, social desirability might have led to more positive answers, noticeably with regards to stigmatization, for instance by admitting only to prosocial behaviour. While several respondents complained of having experienced stigmatization in the context of hearing health, only one participant actually displayed stigmatizing behaviour (i.e., the comment on choosing a desk job if one cannot deal with the sound pressures in an orchestra). It is therefore likely that the answers provided in this study are more positive and show more socially acceptable or expected behaviour than would be the case in all orchestra musicians.

Lastly, as the qualitative answers were given in response to open questions rather than interviews, there was no opportunity to check back with the participants as to the meaning of a particular phrase or to ask for more explanations or examples for certain answers. Thus, while the qualitative data offer unique insights into the reasoning and concerns of professional and amateur musicians regarding their hearing health, future studies should apply interviews or focus groups, for instance, to gain a more detailed understanding of the topic.

In summary it can be stated that while musicians feel that their hearing health is important, they do not regularly take measures to prevent hearing issues. The present study found a need for audiological personnel to be trained on advising musicians regarding hearing protection, as well as more information provided to musicians on this topic – be it by orchestras, health insurances, or other health providers.

## Methods

### Participants

This study included a total of 771 participants: 401 amateur (mean age = 48.9 years, SD = 19.1; 232 female, 1 non-binary, 18 not specified) and 370 professional orchestral musicians (mean age = 48.4 years, SD = 11.8, 164 female, 1 non-binary, 30 non-specified). The vast majority of amateur musicians were affiliated with concert orchestras (81%) or multi-genre orchestra (4%). Among professionals, the majority were affiliated with theatre/opera orchestras (36%), followed by concert (26%), multi-genre (23%), and radio orchestras (13%). The remaining individuals (both professionals and amateurs) selected the “others” category.

### Materials

#### Questionnaire

The original survey instrument comprised a comprehensive set of 127 questions designed to assess several key areas within the context of orchestral musicianship. These included demographics (age, gender, instrument, orchestra type), sound level exposure, hearing ability, hearing protection practices, use of technical hearing aids, general satisfaction, and health-related quality of life. However, the current paper focuses specifically on the hearing awareness and hearing protection components of the survey data. To assess hearing ability, participants were asked to report the results of their prior audiological assessment. Those without such data completed an adapted version of the Hearing Aid for Music (HAfM) questionnaire^38^. Hearing loss was then categorized using a modified WHO^39^ classification system, incorporating criteria from Martini^40^ to capture milder forms of impairment.

### Procedure

This cross-sectional survey was conducted between February 2022 and February 2023. Participants were recruited through a multifaceted approach, including collaboration with the German Orchestra Association’s internal newsletter, direct email invitations to amateur orchestras across Germany and Austria, and outreach to orchestral management and choirs (for distribution details, see Hake et al. [^36^]). This research, however, focuses specifically on orchestral musicians. The survey was constructed using SoSci Survey (v.3.2.44^41^) and was offered in English and German. Participants completed the survey anonymously and in their own time. Furthermore, participants had the opportunity to skip questions. On average, the completion time was 20 min. Ethical approval was granted by the Carl von Ossietzky Universität Oldenburg ethics review board (Drs.EK/2021/114) and all experiments were performed in accordance with relevant guidelines and regulations. Informed consent was obtained from all participants. As no identifying information was gathered from the participants, no consent for publication of this information was necessary.

### Data analysis - quantitative

No data cleaning has been done prior to the analysis. Incomplete or partially filled questionnaires were retained during data analysis to preserve potentially valuable insights from the qualitative responses. Thus, sample sizes (N) may vary across analyses and are explicitly stated. Given the ordinal data structure, non-parametric statistical tests were selected. Specifically, the Mann-Whitney U tests (also called the Wilcoxon Rank-Sum Test) was employed to test for group differences. To assess the effect size of the non-parametric Mann-Whitney U test (refered as *W*), the rank-biserial correlation (*r*_*rb*_) was calculated. Generally, a *r*_*rb*_ closer to zero indicates a weak relationship, values around 0.5 indicate a moderate relationship, and values close to 1 indicate a very strong relationship. To account for multiple comparisons, the Bonferroni correction was applied. All statistical analyses were conducted using R statistical software (version 2022.07.2 + 576;^42^).

### Data analysis – qualitative

A range of open questions were included in the questionnaire to ask for more specific details and anything the participants wanted to comment on. All answers that were given were considered for the qualitative analysis. Overall, 621 orchestra musicians (leaders, professionals and amateurs) had provided responses.

Qualitative analysis was carried out following Kuckartz (ch. 5)^43^. A category system was created based on the topics of each question. In some cases, for instance, hearing protection or hearing aids, several questions related to the same topic and thus a broader (deductive) category encompassing all associated questions was created. Once each broad category was agreed on among the research team, a system of subcategories was created by ES, which had the additional benefit of dividing the answers into more manageable chunks. The coding was then checked by MB and DD and, applying consensual coding, all disagreements (e.g., subcategory names or which code belonged into which category) were then resolved among these three authors.

## Data Availability

The data from this study has been made publicly accessible via the GESIS research data management platform. It can be accessed online at the provided URL [https://doi.org/10.7802/2695] (see Hake et al. [36]).
